# 3D visualisation of hepatitis B vaccine in the oral delivery vehicle SBA-15

**DOI:** 10.1038/s41598-019-42645-5

**Published:** 2019-04-15

**Authors:** Martin K. Rasmussen, Nikolay Kardjilov, Cristiano L. P. Oliveira, Benjamin Watts, Julie Villanova, Viviane Fongaro Botosso, Osvaldo A. Sant’Anna, Marcia C. A. Fantini, Heloisa N. Bordallo

**Affiliations:** 1 Niels Bohr Institute, University of Copenhagen, Copenhagen, Denmark; 2 Department of Health Technology, Technical University of Denmark, Kongens Lyngby, Denmark; 3 Helmholtz Center Berlin for Materials and Energy, Berlin, Germany; 4 Institute of Physics, São Paulo, Brazil; 5 Paul Scherrer Institute, Villigen, Switzerland; 6 ESRF-The European Synchrotron, ID16B Grenoble, France; 7 Virology Laboratory, Butantan Institute, São Paulo, Brazil; 8 Immunochemistry Laboratory, Butantan Institute, São Paulo, Brazil; 9 European Spallation Source (ESS), Lund, Sweden

## Abstract

Developing a technology that enables oral vaccines to work efficiently remains a considerable effort since a number of difficulties must be addressed. The key objective being to ensure the safe passage through the harsh conditions within the gastrointestinal tract, promoting delivery that induces enhanced immune response. In the particular case of hepatitis B, the oral formulation in the nanostructured silica SBA-15 is a viable approach. As a result of its porous structure, low toxicity and structural stability, SBA-15 is capable to protect and release the hepatitis B surface antigen (HBsAg), used in the vaccination scheme, at the desired destination. Furthermore, when compared to the currently used injection based delivery method, better or similar antibody response has been observed. However, information about the organisation of the antigen protein remains unknown. For instance, HBsAg is too large to enter the 10 nm ordered mesopores of SBA-15 and has a tendency to agglomerate when protected by the delivery system. Here we report on the pH dependence of HBsAg aggregation in saline solution investigated using small angle X-rays scattering that resulted in an optimisation of the encapsulation conditions. Additionally, X-ray microscopy combined with neutron and X-ray tomography provided full 3D information of the HBsAg clustering (i.e. agglomeration) inside the SBA-15 macropores. This method enables the visualisation of the organisation of the antigen in the interior of the delivery system, where agglomerated HBsAg coexists with its immunological effective uniformly distributed counterpart. This new approach, to be taken into account while preparing the formulation, can greatly help in the understanding of clinical studies and advance new formulations.

## Introduction

Hepatitis B, a viral disease causing infection in the liver, is most efficiently prevented by vaccination. The current and highly efficient injection based vaccine uses the aluminium salt, Al(OH)_3_, as an adjuvant to increase the immunological response. Although the aluminium based vaccine is considered safe, it can cause side effects such as swelling, inflammation and abscess at the injection site^1^. On the other hand oral vaccination offers many advantages, including decreased side effects, easy administration and substantially reduced costs^2–5^. Therefore, developing delivery vehicles capable of protecting the vaccine from the harsh gastric environment and performing delivery in the intestine to stimulate antibody production is desireable.

The nanostructured mesoporous silica SBA-15, formed by ~20 μm particles with hexagonal ordered mesopores with a diameter of 10 nm and macropores larger than 50 nm, is especially interesting^6–11^. The advantages of SBA-15 are its low toxicity and structural stability in biological environments which prevents premature release^9,12,13^. SBA-15 has been shown to be an efficient carrier, despite hepatitis B surface antigen (HBsAg), the protein used in the vaccination scheme, being too large (22 nm) to enter the mesopores^7,14,15^. Indeed, recent *in vivo* mice studies have reported that SBA-15 induces better or similar immunological response compared to the traditional injection based vaccination method, as after immunisation a higher level of antibodies and immune cells are observed^7^. Another important consideration is the fact that the macrophages can recognize size and shapes of their targets and in the case of SBA-15 neither cell integrity nor phagocity potential are affected^16^. Previous assays clearly showed that SBA-15 is engulfed by macrophages while keeping cell viability^17–19^. On the other hand physical chemical characterisation using small angle X-ray scattering (SAXS) showed that the protein has a tendency to agglomerate (hereafter used to describe clustering of the encapsulated HBsAg) inside SBA-15^7^, which would severly hinder the induced production of antibodies^20,21^.

In this work we hypothesise that agglomerated and non-agglomerated HBsAg are protected in the macropores and that agglomeration occurs only after encapsulation. With SAXS and dynamic light scattering (DLS) we demonstrate that in saline solution HBsAg is indeed a very stable protein that does not show significant tendency to aggregate, i.e. form clusters before encapsulation, with 7.4 being the optimal pH for lessening this effect. Analysis of the state of encapsulated proteins is, however, a very challenging task; traditionally only achieved indirectly by techniques such as thermal analysis, diffraction, TEM, NMR spectroscopy, light scattering and spectroscopy^22–27^. Here, by combining the penetrating and non destructive properties of neutrons and the high spatial resolution offered by X-ray imaging, direct spatial information of HBsAg distribution inside the SBA-15 particles was obtained. Neutrons interact with the nuclei giving information on proton distribution, thereby allowing direct observation of the hydrogen atoms of the protein within the carrier, while X-rays provide information on heavier elements present in NaCl and SiO_2_. Thus using X-ray and neutron tomography, we verified that for different HBsAg to SBA-15 mass ratio, the antigen and its buffer were observed together, but non-uniformly distributed. These measurements demonstrated that HBsAg agglomerates the least upon encapsulation in the mass ratio 1:40, making this the optimal condition for oral delivery. Additionally, X-ray phase contrast tomography verified that some SBA-15 particles contain agglomerated antigen, leaving space for its immunologically effective, non-agglomerated counterpart, in the remaining macropores. Finally, by means of scanning transmission X-ray microscopy (STXM) and near edge X-ray absorption fine structure (NEXAFS), non-agglomerated uniformly distributed protein was clearly detected in the macropores of all analysed SBA-15 particles. An important application of this approach during the formulation process is the ability to control the distribution of the protein in the delivery vehicle as well as to clarify if the efficiency of oral vaccines is directly related to the morphology of the carrier.

## Results

### HBsAg in solution

**Figure 1:**
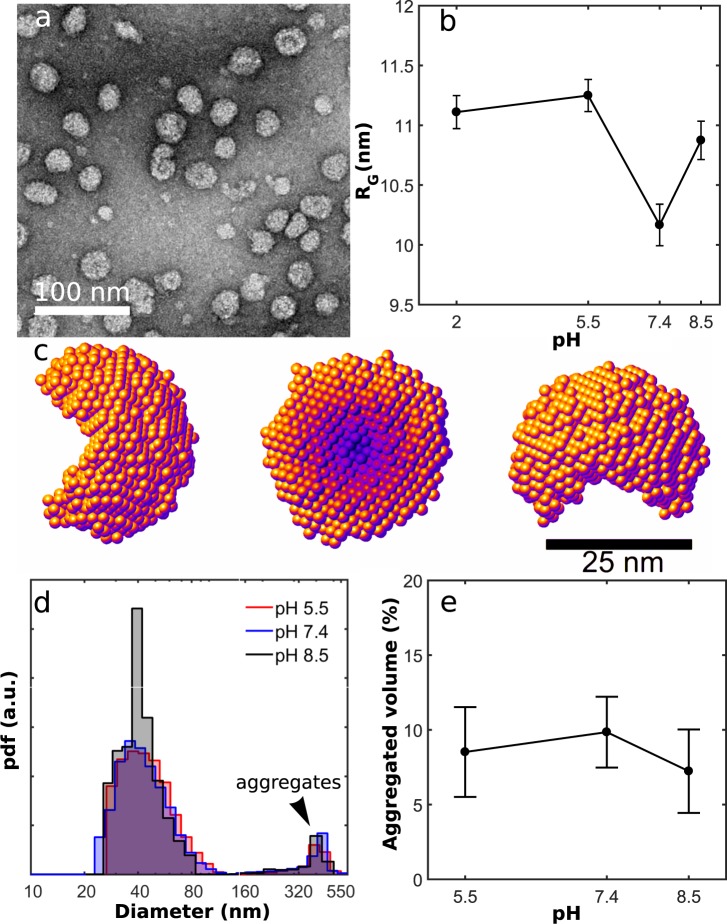
(**a**) Transmission electron microscopy (TEM) image of HBsAg particle with diameters in the range 25 nm to 35 nm. (**b**) Radius of gyration *R*_*G*_ of HBsAg as a function of pH showing a minimum at 7.4 and (**c**) The morphology HBsAg obtained using ab initio modeling of the SAXS data. (**d**) Diameter distribution of HBsAg by volume showing that HBsAg forms aggregates larger than 200 nm and (**e**) Relative HBsAg volume in large aggregates as a function of pH showing no clear dependence, as seen by DLS.

HBsAg, prepared in phosphate buffered saline solution (PBS, 10 mM Na_2_HP0_4_), (Fig. 1a) at different pHs, was characterized by small angle X-ray scattering (SAXS) which provides information on the distribution of particles up to ~40 nm in diameter. By analysing the data, provided in Sup. Fig. 1, with the Inverse Fourier Transform (IFT) method^28^ pair distance distributions (PDD) for the HBsAg particle showed a stable morphology for all pH environments (Sup. Fig. 1). From the PDD results the particle shape was modelled as an unclosed sphere with a diameter of (32 ± 4) nm (Fig. 1c), which is too large to enter the 10 nm mesopores of SBA-15. The results were obtained by five *DAMMIN* runs which were averaged using the *DAMAVER* software ATSAS package^29^. Furthermore, the radius of gyration *R*_*G*_ as a function of pH was calculated (Fig. 1b), and a minimum at pH 7.4 was observed. Therefore it was concluded that this value is the optimal pH for avoiding protein aggregation before encapsulation.

In a second step, to analyse if these samples contained larger aggregates dynamic light scattering (DLS) was performed. The DLS results weighted by the number of particles (Sup. Fig. 2) showed that the hydrodynamic diameter of HBsAg was ~30 nm in the measured pH range. This value is consistent with the hydrodynamic diameter (30 ± 3) nm calculated from the shape model obtained from the SAXS data, using the software package *HydroPro*^30^. Additionally, Fig. 1d depicts the HBsAg diameter distribution by volume, also obtained from the DLS analysis using the NNLS method^31^. Aggregates with diameters larger than 200 nm are formed for all pH values, Fig. 1e. From these measurements we conclude that while the radius of gyration of the average HBsAg particle has a minimum at pH 7.4, making this value optimal for encapsulation of the antigen, prevention of a small amount of large protein aggregates can not be completely avoided.

### Visualizing HBsAg encapsulated in SBA-15

**Figure 2:**
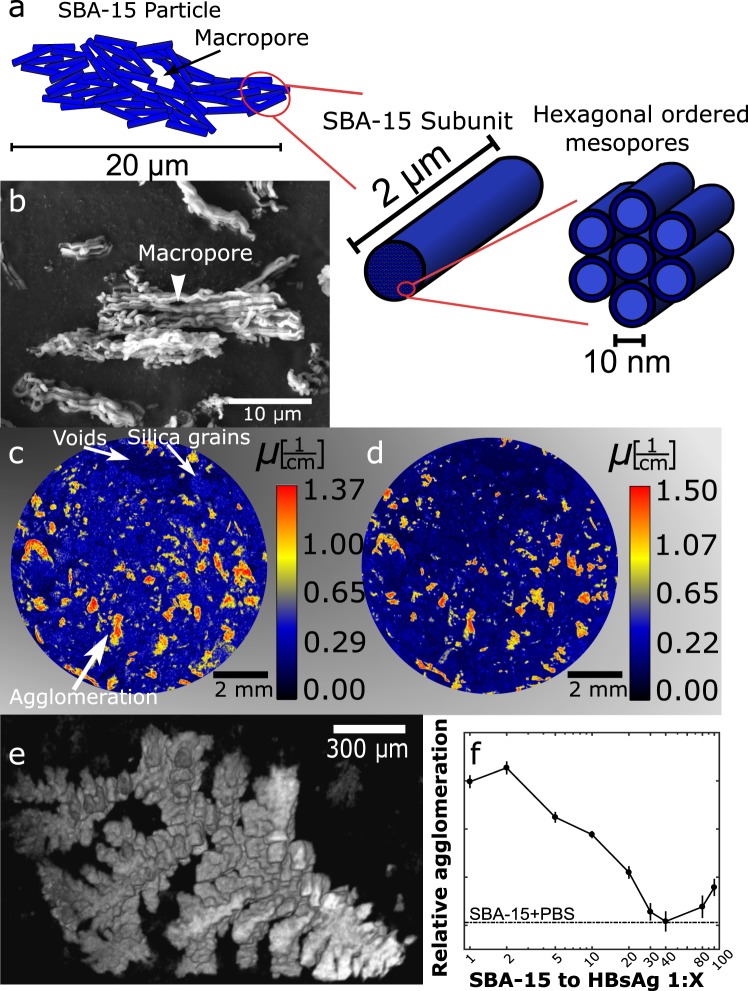
(**a**) Schematic of the SBA-15 structure, showing the 20 μm particles forming macropores larger than 50 nm in between the 2 μm rods subunits, with hexagonal ordered mesopores having a diameter of 10 nm. (**b**) Scanning electron microscopy (SEM) image of SBA-15 particles with few macropores visible from the surface. (**c**) Neutron tomogram, obtained using the CONRAD-2 beamline, of a cross sectional slice of HBsAg encapsulated in SBA-15 for the mass ratio 1:2. The protein agglomerates forming large non evenly distributed structures in the SBA-15 grains. (**d**) X-ray tomogram aligned with (**c**) showing PBS agglomerated with the large protein structures. (**e**) 3D visualisation of a typical HBsAg agglomeration occurring inside a SBA-15 grain. (**f**) Relative amount of HBsAg agglomeration as a function of HBsAg:SBA-15 mass ratio varying from 1:1 to 1:100. For the ratio HBsAg:SBA-15 1:40 agglomeration is so small that it is indistinguishable from PBS alone.

To obtain 3D information of the antigen protein at pH 7.4 encapsulated in the SBA-15 structure (Fig. 2a,b) using the procedure described in^6^, neutron attenuation tomography was performed at the CONRAD-2 instrument at the BER II research reactor^32,33^. From a cross section slice, Fig. 2c, we can distinguish the weakly attenuating silica grains from empty voids in the powder sample. We also observe large areas of agglomerated HBsAg that are indicated by the high attenuation of the neutrons caused by the hydrogen atoms in the protein and its hydration shell. To probe the spatial distribution of the salt solution X-ray attenuation tomography was performed using the same pixel size of 6.37 μm as for the neutron measurements. This allowed us to align the images for direct comparison. Figure 2d shows a X-ray tomogram of the same part of the sample shown in (c), high attenuation is observed at the same positions and with similar structures in both datasets. Thus from these results we conclude that HBsAg and PBS form large agglomerates within the grains of SBA-15, and that the agglomerated HBsAg is not uniformly distributed. On the other hand, the control samples, i.e. PBS incorporated into SBA-15 without antigen, only showed small agglomeration with drastically different morphology (Sup. Fig. 3), than the branching out HBsAg agglomerations 3D visualised in Fig. 2e, for the sample SBA-15 1:2. Consequently we can argue that HBsAg tends to form large agglomerations.

After setting a pixel threshold value for the agglomeration, the tomography images were used to analyse the most efficient HBsAg to SBA-15 mass ratio. Figure 2f shows the relative agglomeration normalised to the amount of encapsulated HBsAg for different HBsAg to SBA-15 mass ratios with constant PBS value. A minimum is observed for the ratio HBsAg:SBA-15 1:40, in good agreement with previous data^7^. For this ratio we observe that the amount of agglomeration is indistinguishable from the control sample, i.e. containing only salt. Therefore only a small amount of the encapsulated HBsAg is clustering in the immunological inactive agglomerations.

**Figure 3:**
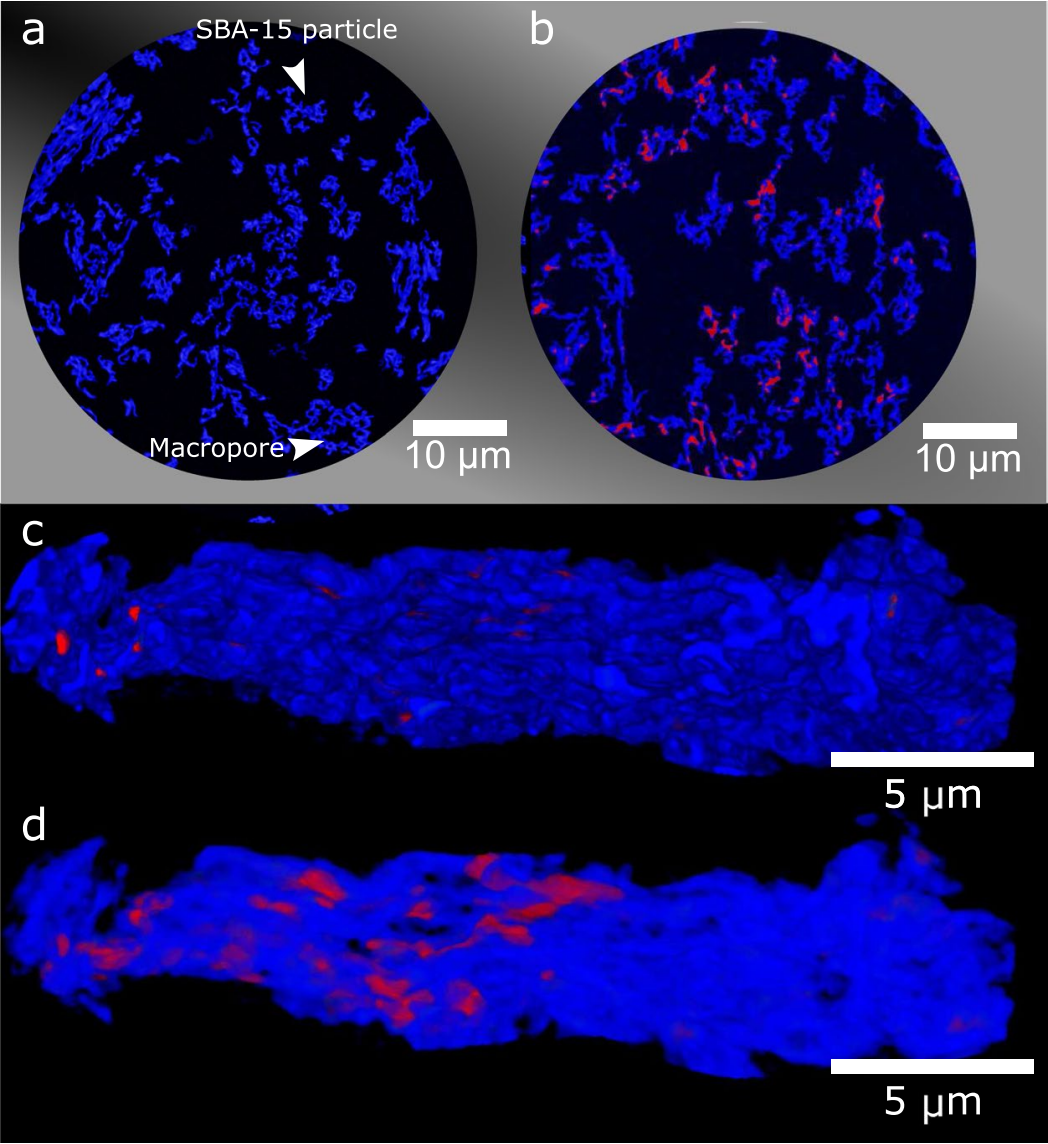
(**a**) X-ray tomogram, obtained at ID16B, of SBA-15, showing the macropores in the SBA-15 particles. (**b**) X-ray tomogram of SBA-15 with encapsulated HBsAg shown to agglomerate in the macropores. (**c**) 3D visualization of SBA-15 with HBsAg, showing small amounts of HBsAg at the surface, (**d**) Same particle as in (**c**), shown as semitransparent allowing visualising the agglomerated protein inside the macropores.

While neutron and X-ray attenuation tomography revealed the morphology and non-uniform distribution of HBsAg agglomeration on the size scale of the SBA-15 grains, X-ray phase contrast tomography with a spatial resolution of 100 nm was requried to obtain 3D information on the SBA-15 particle level (~20 μm, sketched in Fig. 2a). These experiments were performed at the ID16B Nano-Analysis beamline at the European Synchrotron Radiation Facility^34^. Figure 3a shows a typical cross section slice of pure SBA-15, where the particles are shown in blue and the dark areas depict voids in the low density silica. This high spatial resolution allows for the visualisation of individual macropores in the SBA-15 particles. The tomogram of SBA-15 with HBsAg, Fig. 3b, shows the presence of agglomerated HBsAg (red) in some macropores. Agglomerated protein is clearly non-uniformly distributed in the particles, while several particles without agglomerations were also observed. From the obtained tomograms it was possible to 3D visualise individual SBA-15 particles. Figure 3c shows a typical particle with only a small amount of HBsAg present on the surface. By making the particle semitransparent, Fig. 3d, we highlight the HBsAg protected in the macropores inside the SBA-15 particle. Analysis of several SBA-15 to HBsAg mass ratio confirmed that the ratio HBsAg:SBA-15 1:40 induces the lowest relative agglomeration of HBsAg (Sup. Fig. 4).

### Immunological active HBsAg in SBA-15

**Figure 4:**
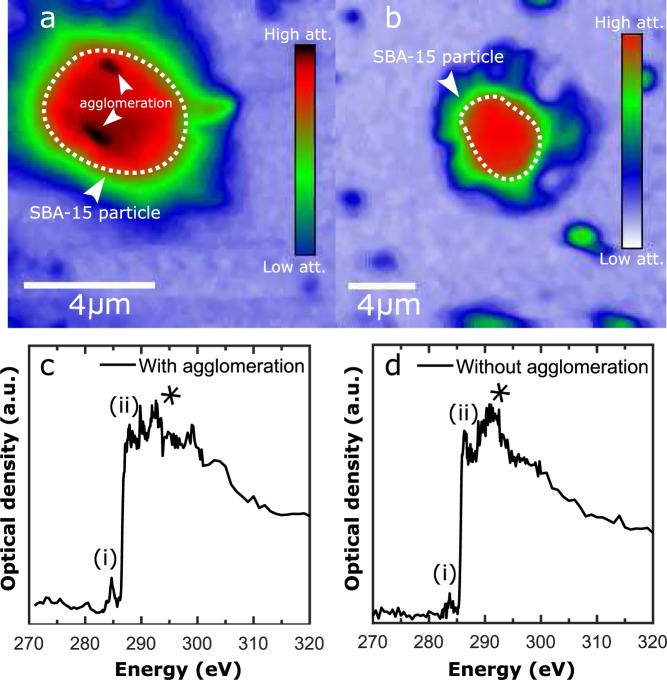
(**a**) STXM image taken with photon energy of 390 eV (above the carbon K-edge 290 eV), at PolLux, of SBA-15 particle with two distinct agglomerations and (**b**) with encapsulated HBsAg without agglomeration. (**c**,**d**) NEXAFS spectrums of the two particles in (**a**,**b**) for energies around the carbon edge showing the presence C=C bonds (i), C=O bonds (ii) and a variety of C-H and C-N bonds (*) attributed to the presence of protein in the particles.

To investigate the presence of non-agglomerated HBsAg in individual SBA-15 particles, scanning transmission X-ray microscopy (STXM) and near edge X-ray absorption fine structure (NEXAFS) were performed at the PolLux instrument at the Swiss light source^35–37^. 2D STXM images with spatial resolution of 80 nm and photon energy of 390 eV, sensitive to the presence of carbon as it is above the K absorption edge (290 eV), are shown in Fig. 4a,b for a SBA-15 particle with and without agglomerated HBsAg, respectively. Both particles attenuate the X-ray beam more than the control particles, i.e. SBA-15 only with PBS (Sup. Fig. 5). NEXAFS line scans performed in the core of the two particles containing HBsAg, depicted in Fig. 4c,d, clearly show the presence of structural carbon. The peak labelled (i) is attributed to the presence of C=C bonds, (ii) arises from C=O bonds^38,39^, while the indistinguishable peaks labelled (*) in the range 287 eV to 300 eV originate from C-H and N-H bonds^40^. These peaks are attributed to the presence of antigen in the SBA-15 particles and were observed for all SBA-15 particles analysed across multiple mass ratios. No agglomerations are observed for the particle in Fig. 4b, demonstrating that the encapsulated HBsAg is also found in a non-agglomerated state, which can induce the immunological response.

## Discussion

This study demonstrates the potential of combining neutron and X-ray imaging techniques with X-ray microscopy to visualise the distribution of the protein antigen HBsAg used for hepatitis B vaccination in the oral delivery vehicle SBA-15. The obtained quantitative 3D structural data was crucial to determine how the non-agglomerated protein is distributed inside SBA-15. Furthermore, it allowed to verify the hypothesis that the silica particles acts on the physical protection of the antigens preventing the protein degradation in the gastric fluid inducing the release in the intestine, where the immune competent cells are found^7^. This information plays an important role in the optimisation process of the antigen encapsulation conditions in SBA-15, essential to obtain higher levels of humoral immune response.

Structural alterations of the HBsAg antigen in solution, previous to encapsulation, were analysed using SAXS and DLS. Ab initio modelling of the SAXS data of HBsAg protein in solution resulted in a half sphere with a diameter of 32 nm, confirming that the antigen is too large to enter the 10 nm mesopores of SBA-15 and therefore must be protected in the macropores. These results suggest that SBA-15 also could be promising as an oral delivery vehicle for other large agents. Through DLS we showed that 3–10% of the HBsAg volume in solution formed larger aggregates. Furthermore, these measurements revealed that the protein is very stable when the sample pH is varied. Interestingly, it was observed that HBsAg at pH 7.4, the condition used for encapsulation, is indeed the optimal value for avoiding formation of undesired aggregates.

3D information of HBsAg encapsulated in SBA-15 at pH 7.4 was obtained using X-ray and neutron attenuation tomography. After careful alignment of the images, it was shown that PBS (used to prepared the encapsulated vaccine) and HBsAg agglomerates together in the powder grains of SBA-15. Non-uniformly distributed agglomerations of the same morphology were observed for all ratios of HBsAg to SBA-15. Additionally phase-contrast tomography showed that PBS and HBsAg agglomerates in the macropores larger than 50 nm, which can easily uptake and protect the 32 nm in diameter antigen particles. By normalising the volume of the agglomerations in SBA-15 to the amount of encapsulated HBsAg, it was confirmed that the ratio for which HBsAg agglomerated the least is 1:40. As agglomerated antigen negatively influences the immunological response, these findings are vital in providing insight for the optimal value used for vaccination^20^. Finally, STXM and NEXAFS analysis clearly showed that non-agglomerated HBsAg is well distributed in all SBA-15 particles. These findings confirm the applicability of the synthesised SBA-15 as a potential delivery vehicle for future developments in oral vaccination.

To conclude, the results obtained with HBsAg encapsulated antigen are the first to demonstrate how SBA-15 protects the antigen for oral administration of the hepatitis B vaccine. Encapsulated vaccines combine the advantages of immunisation with the ease of administration by the oral route and concomitant induction of the immune response. As shown here, 3D imaging techniques open new paths to overcome the challenges in the development of oral vaccination as it allows direct visualisation of the several components. Being able to map the antigen distribution within the carrier, leads to crucial information needed for a complete understanding of the results from biological assays.

## Methods

### Sample preparation

HBsAg samples with a concentration of 0.45 $\frac{{\rm{mg}}}{{\rm{mL}}}$, measured by ultra violet absorption, was prepared at pH 5.5, 7.4 and 8.5 in a phosphate buffered saline solution (PBS, 10 mM Na_2_HP0_4_). HBsAg at pH 7.4 was encapsulated in a commercially produced SBA-15, synthesized as described in^6^, by dissolving 250 mg of SBA-15 in the 0.45 $\frac{{\rm{mg}}}{{\rm{mL}}}$ HBsAg solution supplied with extra PBS (10 mM Na_2_HP_4_) to obtain a final volume of 500 mL. By changing the initial volume of HBsAg solution, samples with different HBsAg to SBA-15 mass ratio ranging from 1:1 to 1:100 were obtained, while the amount of salt was kept constant. After mixing, the samples were dried at 35 for 24 hours to obtain a powder of encapsulated protein.

### Experimental methods

Small angle X-ray scattering (SAXS) was performed using a Xenocs Xeuss set-up with a Pilatus bidimensional detector and a wavelength of *λ* = 0.15418 nm. HBsAg in solution at different pH, ranging from 2.0 to 8.5, was mounted in capillaries with a diameter of 1 mm. Measurements were performed for PBS buffer and water, as background and reference to obtain the absolute scattering intensity^41^.

Dynamic light scattering (DLS) analysis for the same samples was performed on a Brookhaven DM-5000 Particle Size Analyzer, using a wavelength of 635 nm.

Neutron attenuation tomography, with a pixel size of 6.37 μm was performed at the cold neutron instrument CONRAD-2 at the BER II research reactor, Helmholtz Zentrum Berlin (HZB, Berlin, Germany), using a 2k × 2k CCD neutron camera detection system. Powder samples with different HBsAg to SBA-15 mass ratios, were mounted in 4 mm high, 10 mm diameter aluminium sample holders for both neutron and X-ray tomography measurements.

X-ray attenuation tomography was performed on the same samples using a *μ*CT operated at 100 keV and 100 μA. A 2k × 2k amorphous-Si flat panel detector allowed for obtaining images with a pixel size of 6.37 μm making the obtained neutron and X-ray tomograms directly comparable.

X-ray phase contrast imaging was performed at the ID16B Nano-analysis beamline at the European Synchrotron Radiation Facility (ESRF, Grenoble, France). Powder samples at selected mass ratios were mounted in a capillary with a diameter of 400 μm. The measurements were performed using a photon energy of 17 keV and a Frelon 4 M camera obtaining a spatial resolution of 100 nm.

Scanning transmission X-ray microscopy (STXM) and near edge X-ray absorption fine structure (NEXAFS) was performed, for selected mass ratios, at the PolLux beamline (PSI, Villigen, Switzerland) at the Swiss Light Source. Both STXM and NEXAFS were performed around the carbon absorption edge (290 eV) with energy resolution of 0.5 eV, on an individual part of SBA-15 particle scale with 80 nm resolution.

## Supplementary information

Supplementary material
