# Supplementary material for: 3D visualisation of hepatitis B vaccine in the oral delivery vehicle SBA-15
Source: Sci Rep. 2019 Apr 15;9:6106. doi: 10.1038/s41598-019-42645-5 (PMC6465313; doi:10.1038/s41598-019-42645-5)
Supplement: Supplementary file 1 — Supplementary material [file 41598_2019_42645_MOESM1_ESM.pdf]

Supplementary material of 3D visualisation of hepatitis B vaccine in the oral delivery vehicle SBA-15

Martin K. Rasmussen[1,2], Nikolay Kardjilov[3], Cristiano L.P. Oliveira[4], Benjamin Watts[5], Julie Villanova[6], Viviane Fongaro Botosso[7], Osvaldo A. Sant'Anna[8], Marcia C.A. Fantini[4], and Heloisa N. Bordallo[1,9]

[1]Niels Bohr Institute, University of Copenhagen, Copenhagen, Denmark
[2]Department of Health Technology, Technical University of Denmark, Kongens Lyngby, Denmark
[3]Helmholtz Center Berlin for Materials and Energy, Berlin, Germany
[4]Institute of Physics, São Paulo, Brazil
[5]Paul Scherrer Institute, Villigen, Switzerland
[6]ESRF-The European Synchrotron, ID16B, Grenoble, France
[7]Virology Laboratory, Butantan Institute, São Paulo, Brazil
[8]Immunochemistry Laboratory, Butantan Institute, São Paulo, Brazil
[9]European Spallation Source (ESS), Lund, Sweden

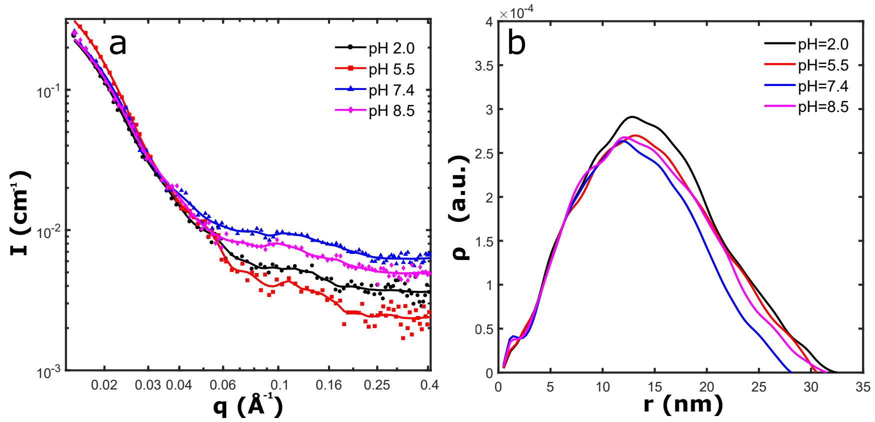

Sup. Fig. 1: **a** SAXS data and Inverse Fourier Transform methods fits for HBsAg in different pH. **b** Obtained pair distance distributions (PDD), showing that the morphology of protein is stable for different pH.

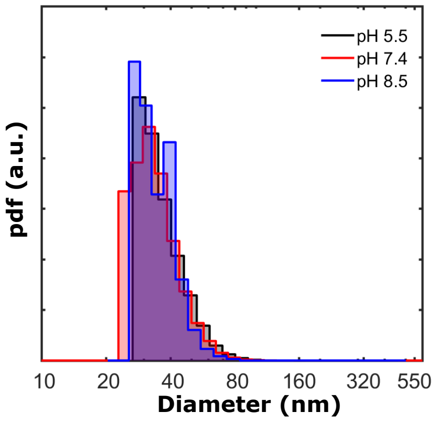

Sup. Fig. 2: Distribution of HBsAg diameters by particle number measured by DLS, at different pH values.

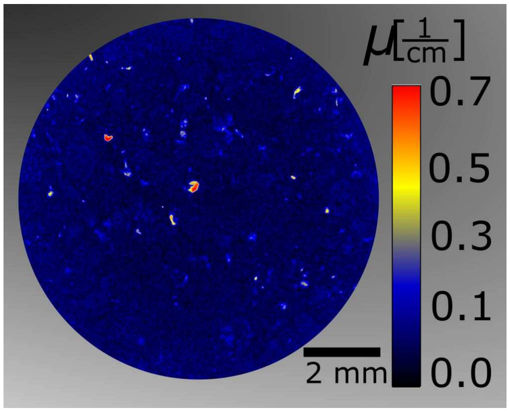

Sup. Fig. 3: Neutron tomogram obtained at the CONRAD beamline of control sample, SBA-15 with PBS, showing few agglomerations.

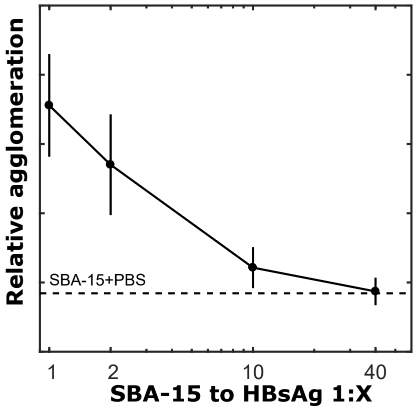

Sup. Fig. 4: Relative amount of agglomerated protein as a function of HBsAg:SBA-15 mass ratio, obtained from phase contrast tomography at ID16B. For the ratio 1:40 the salt and protein agglomeration amount is indistinguishable from PBS alone.

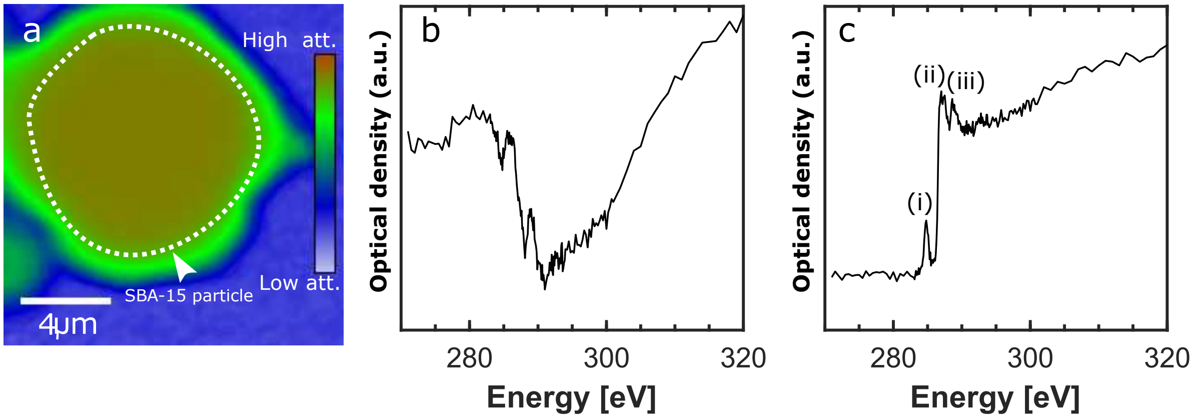

Sup. Fig. 5: **a** STXM image of SBA-15 of a control particle taken with photon energy E = 390 eV. **b** NEXAFS spectrum from the core of SBA-15+PBS particle showing no characteristics of carbon inside the particle. **c** NEXAFS spectrum of carbon contamination around the particle showing the presence C=C bonds (i), C=O bonds (ii) and a clear peak at (iii) attributed to C=C bonds. Data collected at the PolLux beamline.
